# Supplementary material for: Striking Departures from Polygenic Architecture in the Tails of Complex Traits
Source: bioRxiv. 2024 Nov 20:2024.11.18.624155. Preprint. [Version 1] doi: 10.1101/2024.11.18.624155 (PMC11601658; doi:10.1101/2024.11.18.624155)
Supplement: Supplement 1 [file NIHPP2024.11.18.624155v1-supplement-1.pdf]

bioRxiv preprint doi: https://doi.org/10.1101/2024.11.18.624155; this version posted November 20, 2024. The copyright holder for this preprint (which was not certified by peer review) is the author/funder, who has granted bioRxiv a license to display the preprint in perpetuity. It is made available under aCC-BY 4.0 International license.

Supplementary Material

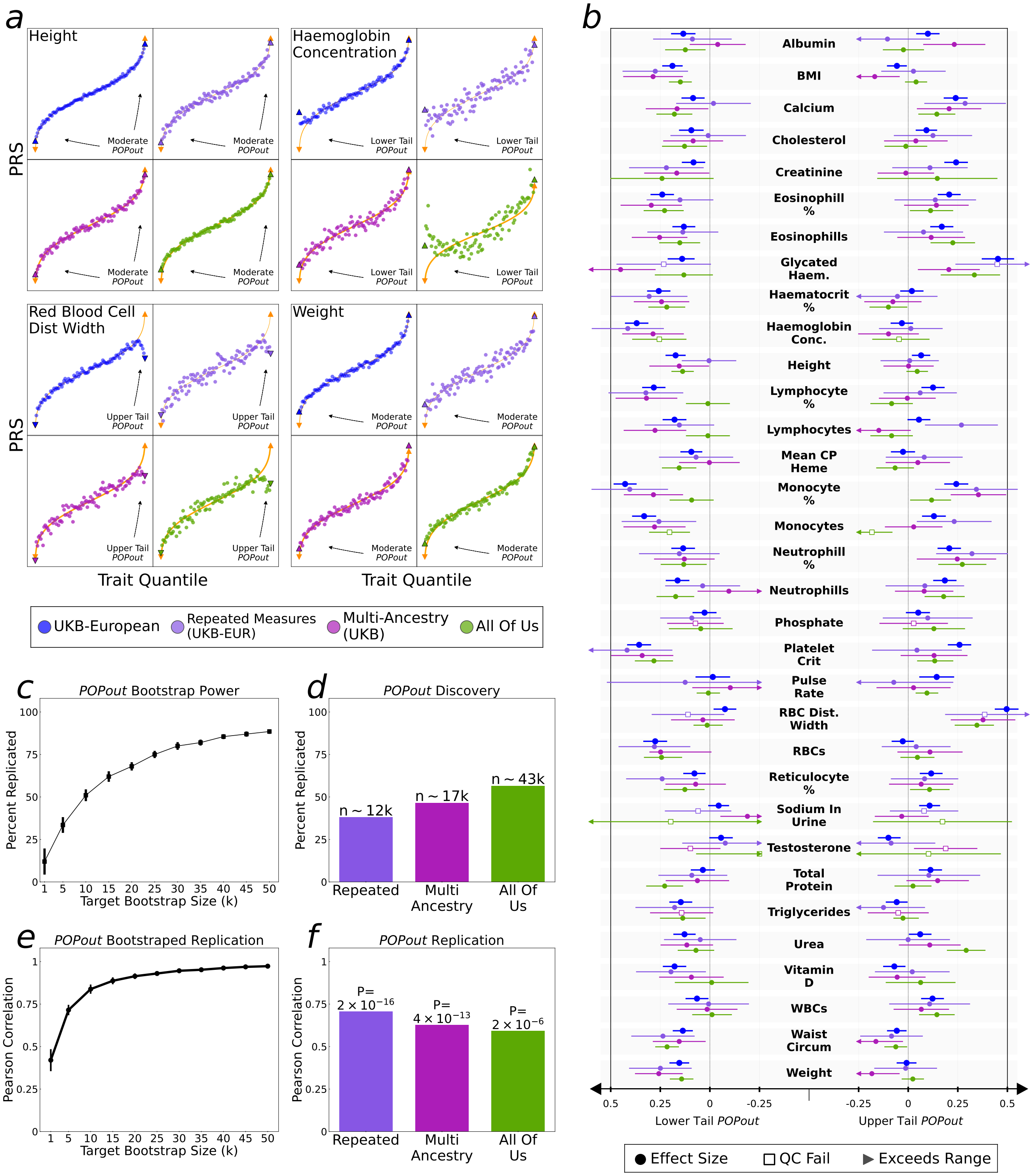

Supplementary Figure 1: Extended Replication a, PRS quantile (in 1% increments) plotted against mean trait value in quantile for four traits with different types of POPout effects in the primary analyses. **b,** POPout effect sizes in primary and replication cohorts across the 33 overlapping traits in the All of Us cohort. **c,d,** Considering trait tails with significant POPout results in our large European ancestry analysis ('Discovery') as true positives, the percent of tails replicated ($P < 0.05$) in 100 bootstrapped runs of different sizes (**c**) and in the replication datasets (**d**). **e,f,** Replication here is defined here as a significant Pearson correlation across effect sizes in our bootstrapped subsets (**e**) and in the replication datasets (**f**).

bioRxiv preprint doi: https://doi.org/10.1101/2024.11.18.624155; this version posted November 20, 2024. The copyright holder for this preprint (which was not certified by peer review) is the author/funder, who has granted bioRxiv a license to display the preprint in perpetuity. It is made available under aCC-BY 4.0 International license.

Trait Info	Trait Statistics	*POPout* Tests *Upper Tail / Lower Tail*	Sib Tests *Upper / Lower*	Replication	Rare/Selection	Figures		
Abbreviated Name [UKB ID],<br>UKB Category,<br>UKB Name,<br>(All of Us Name)	Samples,Sib Pairs<br>SNP-h^2,PRS-r^2<br>Sib-h^2	Pval, 5% FDR, Effect Size, 95% CI	Meta<br>P-values	Repeated Measures | Multi-Ancestry | All Of Us<br>Target Size, QC, pvals, effect sizes (Lower,Upper)	Rare Variants:<br>(Lower, Upper),<br>Model Params:<br>($Y \sim Xo+\beta X+\gamma X^2$)	PRS-on-Trait quantile plots	Conditional Sibling Distribution	Selection Inference Distribution
Age Hay Fever [3761]<br>Questionnaire/Cognitive<br>Age Hay Fever, Rhinitis or Eczema Diagnosed<br>NA	53.9k, 864,<br>8%, 1%<br>32%	0.058,Yes, 0.09, [0.19,-0.0]<br>0.6,Yes, 0.03, [-0.08,0.14]	0.043<br>0.107	2.5k, PASS, (0.505, 0.883), (-0.1, -0.04)<br>4.9k, PASS, (0.316, 0.147), (-0.16, -0.21)<br>NA	0, 1<br>-0.042, 0, 0			
Albumin [30600]<br>Biomarkers<br>Albumin<br>Albumin [M/v] in Serum or Plasma	122k, 9k,<br>10%, 3%<br>29%	5.0e-04,Yes, 0.1, [0.15,0.04]<br>6.8e-06,Yes, 0.13, [0.08,0.19]	0.876<br>0.413	8.6k, PASS, (0.373, 0.335), (0.09, 0.1)<br>15.9k, PASS, (0.574, 0.003), (-0.04, -0.23)<br>73.5k, PASS, (7.2e-04, 0.524), (0.12, 0.02)	2, 8<br>-0.018, 0, -0.004			
Alphanum. Trail [20157]<br>Questionnaire/Cognitive<br>Duration To Complete Alphanumeric Path (trail #2)<br>NA	56.7k, 1k,<br>11%, 1%<br>47%	1.3e-04,Yes, 0.19, [0.29,0.1]<br>0.092,Yes, -0.08, [-0.18,0.01]	0.2<br>0.934	NA<br>2.3k, PASS, (1.0e-07, 0.026), (-1.07, -0.55)<br>NA	0, 0<br>-0.062, -0.013, 0			
Ankle Spacing [3143]<br>Physical Measures<br>Ankle Spacing Width<br>NA	96k, 7k,<br>23%, 6%<br>65%	0.215,Yes, 0.04, [0.1,-0.02]<br>0.168,Yes, 0.04, [-0.02,0.11]	0.371<br>0.873	NA<br>8.3k, FAIL, (0.048, 0.876), (0.23, -0.02)<br>NA	3, 0<br>-0.022, 0, 0			
Apolipoprotein A [30630]<br>Biomarkers<br>Apolipoprotein A<br>NA	121k, 9k,<br>17%, 8%<br>43%	5.5e-06,Yes, 0.13, [0.18,0.07]<br>2.9e-04,Yes, 0.1, [0.05,0.16]	0.263<br>0.357	8.5k, PASS, (0.048, 0.658), (0.22, 0.05)<br>15.9k, PASS, (0.957, 0.007), (-0.0, -0.22)<br>NA	12, 14<br>-0.012, -0.025, -0.009			
BMI [21001]<br>Physical Measures<br>Body Mass Index (bmi)<br>BMI-mean	167k, 17k,<br>17%, 5%<br>49%	0.017,Yes, -0.06, [-0.01,-0.1]<br>2.9e-14,Yes, 0.19, [0.14,0.24]	0.022<br>0.148	15.4k, PASS, (0.001, 0.745), (0.28, -0.03)<br>22.3k, PASS, (1.8e-04, 0.008), (0.29, 0.17)<br>127k, PASS, (1.9e-13, 0.022), (0.21, -0.06)	1, 0<br>0.004, 0.034, -0.025			
BMR [23105]<br>Physical Measures<br>Basal Metabolic Rate<br>NA	165k, 16k,<br>23%, 9%<br>68%	0.211,Yes, 0.03, [0.07,-0.02]<br>1.1e-04,Yes, 0.1, [0.05,0.14]	0.139<br>0.249	15.1k, PASS, (0.415, 0.759), (0.07, -0.02)<br>22.2k, PASS, (2.9e-04, 5.1e-07), (0.28, 0.3)<br>NA	5, 10<br>0.002, 0.042, -0.023			
Birth Weight [20022]<br>Questionnaire/Cognitive<br>Birth Weight<br>NA	95.6k, 6k,<br>8%, 1%<br>70%	0.336,Yes, -0.03, [0.03,-0.1]<br>2.2e-07,Yes, 0.17, [0.11,0.23]	0.088<br>0.038	7.1k, FAIL, (0.002, 0.354), (0.4, 0.11)<br>6.7k, FAIL, (0.356, 0.069), (-0.12, -0.18)<br>NA	2, 0<br>-0.021, 0.011, 0			
Body Fat % [23099]<br>Physical Measures<br>Body Fat Percentage<br>NA	165k, 16k,<br>17%, 5%<br>49%	0.016,Yes, -0.06, [-0.01,-0.1]<br>1.1e-12,Yes, 0.18, [0.13,0.23]	0.06<br>0.065	15.1k, PASS, (0.022, 0.497), (0.19, 0.05)<br>22.2k, PASS, (0.019, 0.991), (0.16, 0.0)<br>NA	1, 0<br>0.005, 0, -0.025			
Body Impedance [23106]<br>Physical Measures<br>Impedance Of Whole Body<br>NA	165k, 16k,<br>21%, 7%<br>54%	0.003,Yes, 0.07, [0.12,0.03]<br>5.2e-06,Yes, 0.11, [0.07,0.16]	0.18<br>0.236	15.1k, PASS, (0.372, 0.282), (0.08, 0.09)<br>22.2k, PASS, (0.518, 0.743), (-0.04, 0.02)<br>NA	5, 10<br>-0.004, -0.045, -0.015			
Calcium [30680]<br>Biomarkers<br>Calcium<br>Calcium [M/v] in Serum or Plasma	121k, 9k,<br>10%, 3%<br>30%	5.0e-16,Yes, 0.24, [0.3,0.18]<br>0.003,Yes, 0.08, [0.03,0.14]	0.437<br>0.022	8.6k, PASS, (0.845, 0.006), (-0.02, -0.29)<br>15.9k, PASS, (0.037, 0.012), (0.17, -0.21)<br>83.4k, FAIL, (7.8e-08, 4.0e-05), (0.18, -0.14)	4, 4<br>-0.019, 0, -0.003			
Cholesterol [30690]<br>Biomarkers<br>Cholesterol<br>Cholesterol [M/v] in Serum or Plasma	133k, 11k,<br>19%, 11%<br>52%	3.9e-04,Yes, 0.09, [0.14,0.04]<br>0.002,Yes, 0.09, [0.03,0.15]	0.097<br>0.007	10.7k, PASS, (0.929, 0.207), (0.01, -0.12)<br>17.4k, PASS, (0.264, 0.63), (0.08, -0.04)<br>64.4k, PASS, (0.006, 0.198), (0.12, -0.05)	7, 11<br>-0.018, 0, -0.005			
Creatinine [30700]<br>Biomarkers<br>Creatinine<br>Creatinine [M/v] in Serum or Plasma	133k, 11k,<br>17%, 6%<br>45%	9.1e-17,Yes, 0.24, [0.3,0.19]<br>0.004,Yes, 0.08, [0.03,0.14]	0.158<br>0.23	10.6k, PASS, (0.021, 0.249), (0.22, -0.11)<br>17.4k, PASS, (0.043, 0.866), (0.17, 0.01)<br>83.9k, PASS, (2.7e-04, 8.8e-23), (0.13, -0.35)	19, 10<br>-0.012, 0.02, -0.01			
Education Age [845]<br>Questionnaire/Cognitive<br>Age Completed Full Time Education<br>NA	109k, 9k,<br>5%, 1%<br>65%	9.9e-07,Yes, 0.15, [0.21,0.09]<br>4.0e-10,Yes, -0.23, [-0.32,-0.15]	4.3e-06<br>0.074	8k, PASS, (0.006, 0.197), (-0.38, -0.14)<br>11.4k, FAIL, (0.12, 1.0e-05), (-0.14, -0.4)<br>NA	0, 0<br>-0.022, -0.044, 0.003			
Eosinophill % [30210]<br>Biomarkers<br>Eosinophill Percentage<br>Eosinophils/100 leukocytes in Blood by AC	129k, 10k,<br>16%, 6%<br>39%	1.7e-13,Yes, 0.21, [0.26,0.15]<br>8.3e-17,Yes, 0.24, [0.18,0.3]	0.021<br>0.036	11.7k, PASS, (0.074, 0.186), (0.15, -0.14)<br>16.7k, PASS, (1.8e-04, 0.082), (0.3, -0.14)<br>61.3k, PASS, (2.5e-12, 0.13), (0.28, -0.06)	2, 12<br>-0.013, 0.02, -0.01			
Eosinophills [30150]<br>Biomarkers<br>Eosinophill Count<br>Eosinophils [#/v] in Blood by AC	129k, 10k,<br>16%, 5%<br>37%	2.1e-09,Yes, 0.17, [0.22,0.11]<br>2.5e-06,Yes, 0.13, [0.08,0.19]	0.102<br>0.322	11.7k, PASS, (0.129, 0.44), (0.14, -0.08)<br>16.7k, PASS, (3.7e-04, 0.177), (0.25, -0.12)<br>60.5k, FAIL, (0.016, 0.991), (0.1, 0.0)	4, 10<br>-0.015, -0.02, -0.007			
FEV Best [20150]<br>Physical Measures<br>Forced Expiratory Volume In 1s Best Measure<br>NA	126k, 9k,<br>17%, 5%<br>48%	0.327,Yes, 0.03, [0.08,-0.03]<br>7.8e-21,Yes, 0.27, [0.21,0.33]	0.283<br>0.009	NA<br>742, PASS, (0.541, 0.795), (0.16, 0.08)<br>NA	1, 4<br>0.0, 0.023, -0.011			
FEV Z-score [20256]<br>Physical Measures<br>Forced Expiratory Volume In 1s Z-score<br>NA	134k, 11k,<br>15%, 4%<br>40%	2.2e-26,Yes, 0.29, [0.35,0.24]<br>0.038,Yes, 0.06, [0.0,0.11]	0.025<br>0.655	NA<br>811, PASS, (0.501, 0.72), (0.24, -0.08)<br>NA	1, 0<br>-0.007, -0.018, -0.006			
FF Trunk Mass [23129]<br>Physical Measures<br>Trunk Fat-free Mass<br>NA	165k, 16k,<br>25%, 10%<br>71%	0.417,Yes, -0.02, [0.03,-0.06]<br>8.7e-14,Yes, 0.18, [0.13,0.23]	0.105<br>0.088	15.1k, PASS, (0.331, 0.152), (0.08, -0.12)<br>22.2k, PASS, (0.063, 0.014), (0.13, 0.15)<br>NA	4, 14<br>-0.002, 0.047, -0.019			
FVC Best [20151]<br>Physical Measures<br>Forced Vital Capacity Best Measure<br>NA	126k, 9k,<br>20%, 6%<br>55%	0.02,Yes, 0.07, [0.12,0.01]<br>9.4e-27,Yes, 0.32, [0.26,0.38]	0.006<br>0.036	NA<br>742, PASS, (0.763, 0.583), (-0.09, -0.06)<br>NA	1, 3<br>0.002, 0.026, -0.013			
Fluid Intellect [20016]<br>Questionnaire/Cognitive<br>Fluid Intelligence Score<br>NA	54.5k, 3k,<br>17%, 2%<br>60%	0.511,Yes, -0.03, [0.05,-0.1]<br>7.7e-04,Yes, 0.14, [0.06,0.22]	0.556<br>0.67	3.6k, PASS, (0.53, 0.218), (-0.1, -0.24)<br>10.3k, PASS, (0.565, 0.02), (-0.06, 0.25)<br>NA	0, 0<br>-0.043, 0, 0			
Glasses Age [2217]<br>Questionnaire/Cognitive<br>Age Started Wearing Glasses Or Contact Lenses<br>NA	143k, 13k,<br>6%, 1%<br>30%	0.044,Yes, -0.09, [-0.01,-0.17]<br>0.51,Yes, 0.01, [-0.03,0.06]	0.035<br>0.016	12.9k, FAIL, (0.272, 0.023), (-0.07, 0.36)<br>17k, PASS, (0.089, 0.47), (0.14, -0.05)<br>NA	0, 2<br>-0.018, 0.054, 0			
Glucose [30740]<br>Biomarkers<br>Glucose<br>NA	121k, 9k,<br>6%, 2%<br>22%	1.1e-13,Yes, 0.22, [0.28,0.16]<br>0.026,Yes, 0.07, [0.03,0.11]	0.005<br>0.439	8.6k, PASS, (0.02, 0.002), (0.27, -0.37)<br>15.8k, PASS, (0.007, 0.136), (0.21, -0.12)<br>NA	2, 3<br>-0.022, -0.015, 0			
Glycated Haem. [30750]<br>Biomarkers<br>Glycated Haemoglobin<br>Hemoglobin A1c/Hemoglobin.total in Blood	127k, 10k,<br>18%, 8%<br>49%	1.6e-50,Yes, 0.45, [0.53,0.37]<br>8.2e-11,Yes, 0.14, [0.08,0.21]	0.001<br>0.165	8.3k, FAIL, (0.053, 4.3e-12), (0.23, -0.45)<br>15.4k, PASS, (9.6e-07, 0.009), (0.45, -0.2)<br>38.9k, PASS, (0.364, 8.9e-09), (0.05, -0.29)	9, 19<br>-0.063, -0.011, 0			
Grip Strength [46]<br>Physical Measures<br>Hand Grip Strength (left)<br>NA	167k, 17k,<br>9%, 2%<br>31%	2.9e-04,Yes, -0.09, [-0.04,-0.14]<br>2.1e-14,Yes, 0.19, [0.14,0.23]	0.064<br>3.8e-05	15.4k, PASS, (0.138, 0.478), (0.13, -0.05)<br>22.5k, PASS, (0.087, 2.7e-04), (-0.1, -0.23)<br>NA	0, 6<br>-0.021, 0.045, 0			

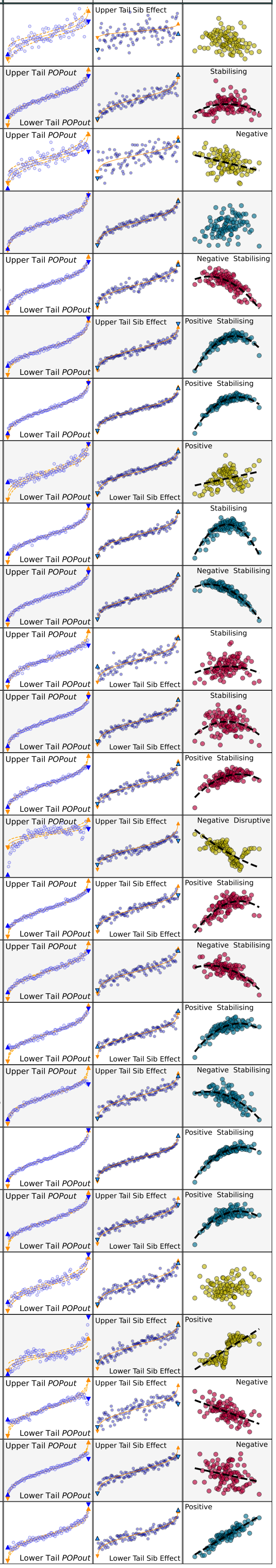

Supplementary Table 1: Traits 1-25

bioRxiv preprint doi: https://doi.org/10.1101/2024.11.18.624155; this version posted November 20, 2024. The copyright holder for this preprint (which was not certified by peer review) is the author/funder, who has granted bioRxiv a license to display the preprint in perpetuity. It is made available under aCC-BY 4.0 International license.

Trait Info	Trait Statistics	*POPout* Tests (Upper Tail / Lower Tail)	Sib Tests (Upper / Lower)	Replication	Rare/Selection	Figures		
Abbreviated Name [UKB ID],<br>UKB Category,<br>UKB Name,<br>(All of Us Name)	Samples,Sib Pairs<br>SNP-h^2,PRS-r^2<br>Sib-h^2	Pval, 5% FDR, Effect Size, 95% CI	Meta<br>P-values	Repeated Measures | Multi-Ancestry | All Of Us<br>Target Size, QC, pvals, effect sizes (Lower,Upper)	Rare Variants:<br>(Lower, Upper),<br>Model Params:<br>($Y \sim Xo + \beta X + \gamma X^2$)	PRS-on-Trait quantile plots	Conditional Sibling Distribution	Selection Inference Distribution
HRT Age [3536]<br>Questionnaire/Cognitive<br>Age Started Hormone-replacement Therapy (hrt)<br>NA	50.1k, 1024,<br>6%, 1%<br>21%	0.441,Yes, 0.05, [0.17,-0.07]<br>0.327,Yes, 0.06, [-0.06,0.17]	0.707<br>0.327	2.5k, PASS, (0.386, 0.555), (-0.15, -0.13)<br>2.1k, PASS, (0.978, 0.642), (-0.0, -0.1)<br>NA	0, 0<br>-0.019, 0, 0			
Haematocrit % [30030]<br>Biomarkers<br>Haematocrit Percentage<br>Hematocrit [Volume Fraction] of Blood by AC	130k, 10k,<br>14%, 4%<br>38%	0.517,Yes, 0.02, [0.07,-0.04]<br>2.9e-19,Yes, 0.26, [0.2,0.31]	0.036<br>0.003	11.7k, PASS, (0.002, 0.591), (0.31, 0.05)<br>16.8k, PASS, (5.9e-04, 0.284), (0.24, 0.08)<br>76k, FAIL, (3.5e-12, 0.002), (0.26, 0.11)	8, 9<br>-0.022, 0.008, 0			
Haemoglobin Conc. [30020]<br>Biomarkers<br>Haemoglobin Concentration<br>Hemoglobin [M/v] in Blood	130k, 10k,<br>15%, 4%<br>40%	0.243,Yes, -0.03, [0.02,-0.09]<br>2.6e-36,Yes, 0.37, [0.31,0.42]	0.155<br>2.3e-05	11.7k, PASS, (1.2e-05, 0.869), (0.41, -0.01)<br>16.8k, PASS, (2.9e-04, 0.195), (0.29, 0.1)<br>83.8k, FAIL, (1.1e-08, 3.8e-05), (0.19, 0.15)	8, 11<br>0.003, 0.005, 0			
Heel BMD [3148]<br>Physical Measures<br>Heel Bone Mineral Density<br>NA	95.8k, 7k,<br>24%, 9%<br>58%	2.5e-16,Yes, 0.29, [0.36,0.22]<br>2.7e-27,Yes, 0.39, [0.32,0.46]	0.003<br>0.002	NA<br>8.3k, PASS, (0.006, 3.7e-06), (0.25, -0.47)<br>NA	2, 3<br>0.003, 0, -0.014			
Heel BUA [3144]<br>Physical Measures<br>Heel Broadband Ultrasound Attenuation<br>NA	95.9k, 7k,<br>22%, 7%<br>51%	3.1e-21,Yes, 0.34, [0.4,0.27]<br>1.2e-16,Yes, 0.29, [0.22,0.36]	0.08<br>0.019	NA<br>8.3k, PASS, (0.192, 1.1e-07), (0.13, -0.47)<br>NA	1, 2<br>0.003, 0.01, -0.014			
Height [50]<br>Physical Measures<br>Standing Height<br>Height-mean	167k, 17k,<br>40%, 21%<br>99%	0.022,Yes, 0.06, [0.02,0.11]<br>1.3e-12,Yes, 0.17, [0.13,0.22]	0.796<br>0.096	15.5k, PASS, (0.95, 0.921), (0.0, -0.01)<br>22.4k, PASS, (0.041, 0.976), (0.15, -0.0)<br>127k, PASS, (2.0e-15, 0.052), (0.22, -0.05)	18, 43<br>-0.002, 0, -0.019			
IGF-1 [30770]<br>Biomarkers<br>Igf-1<br>NA	132k, 11k,<br>20%, 6%<br>49%	0.343,Yes, 0.02, [0.08,-0.03]<br>4.4e-09,Yes, 0.16, [0.11,0.22]	0.419<br>0.219	10.5k, PASS, (0.319, 0.781), (0.09, -0.03)<br>17.3k, PASS, (0.142, 0.038), (0.11, -0.16)<br>NA	10, 12<br>-0.012, -0.008, -0.01			
Imm. RT Frac [30280]<br>Biomarkers<br>Immature Reticulocyte Fraction<br>NA	128k, 10k,<br>11%, 4%<br>31%	4.7e-04,Yes, 0.1, [0.15,0.04]<br>1.5e-15,Yes, 0.21, [0.15,0.26]	0.914<br>0.541	8.2k, PASS, (0.357, 0.894), (0.1, 0.02)<br>16.4k, PASS, (0.023, 0.066), (0.17, -0.14)<br>NA	10, 3<br>-0.022, 0, 0			
Leg Fat % [23115]<br>Physical Measures<br>Left Leg Fat Percentage<br>NA	165k, 16k,<br>16%, 4%<br>48%	0.462,Yes, -0.02, [0.03,-0.06]<br>1.7e-09,Yes, 0.15, [0.1,0.19]	0.647<br>0.335	15.1k, PASS, (0.013, 0.514), (0.2, 0.05)<br>22.2k, PASS, (0.032, 0.797), (0.14, -0.02)<br>NA	1, 0<br>0.005, 0.027, -0.025			
Leg Impedance [23107]<br>Physical Measures<br>Right Impedance Of Leg<br>NA	165k, 16k,<br>19%, 6%<br>54%	0.009,Yes, 0.06, [0.11,0.02]<br>5.2e-11,Yes, 0.17, [0.12,0.22]	0.603<br>8.5e-04	15.1k, PASS, (0.007, 0.357), (0.23, 0.08)<br>22.2k, PASS, (0.053, 0.817), (0.13, -0.02)<br>NA	4, 4<br>-0.007, -0.022, -0.013			
Lymphocyte % [30180]<br>Biomarkers<br>Lymphocyte Percentage<br>Lymphocytes [#/v] in Blood by AC	129k, 10k,<br>13%, 4%<br>35%	9.7e-06,Yes, 0.13, [0.18,0.07]<br>1.1e-22,Yes, 0.28, [0.23,0.34]	0.043<br>0.078	11.7k, PASS, (8.3e-04, 0.514), (0.32, -0.06)<br>16.8k, PASS, (5.9e-05, 0.949), (0.32, 0.0)<br>NA	10, 7<br>-0.014, 0.032, -0.008			
Lymphocytes [30120]<br>Biomarkers<br>Lymphocyte Count<br>Lymphocytes [#/v] in Blood by AC	129k, 10k,<br>17%, 5%<br>44%	0.052,Yes, 0.05, [0.11,-0.0]<br>9.0e-10,Yes, 0.18, [0.12,0.23]	0.177<br>0.808	11.7k, PASS, (0.081, 0.004), (0.15, -0.27)<br>16.8k, PASS, (5.6e-04, 0.067), (0.28, 0.15)<br>61.7k, FAIL, (0.005, 0.621), (0.11, -0.02)	12, 7<br>-0.022, 0.02, 0			
Match ID Time [20023]<br>Questionnaire/Cognitive<br>Mean Time To Correctly Identify Matches<br>NA	166k, 16k,<br>6%, 1%<br>36%	9.9e-07,Yes, 0.12, [0.17,0.07]<br>0.677,Yes, 0.01, [-0.04,0.06]	0.043<br>0.164	15.4k, PASS, (0.411, 0.005), (-0.07, -0.24)<br>21.2k, FAIL, (6.0e-06, 0.005), (0.3, 0.2)<br>NA	1, 0<br>-0.013, -0.026, -0.007			
Mean CP Heme [30060]<br>Biomarkers<br>Mean Corpuscular Haemoglobin Concentration<br>Hemoglobin [M/v] in Blood	130k, 10k,<br>5%, 1%<br>20%	0.369,Yes, -0.03, [0.03,-0.08]<br>4.3e-04,Yes, 0.09, [0.04,0.15]	0.495<br>0.59	11.8k, PASS, (0.457, 0.405), (0.07, -0.08)<br>16.8k, PASS, (0.974, 0.55), (0.0, -0.05)<br>NA	5, 5<br>-0.022, 0, 0			
Mean CP Vol [30040]<br>Biomarkers<br>Mean Corpuscular Volume<br>NA	130k, 10k,<br>23%, 11%<br>54%	2.0e-05,Yes, 0.11, [0.16,0.05]<br>3.0e-97,Yes, 0.44, [0.34,0.55]	0.309<br>5.1e-05	11.8k, FAIL, (1.0e-07, 0.04), (0.51, -0.2)<br>16.7k, FAIL, (0.82, 0.008), (-0.02, 0.21)<br>NA	15, 21<br>-0.022, -0.03, 0			
Mean RT Vol [30260]<br>Biomarkers<br>Mean Reticulocyte Volume<br>NA	128k, 10k,<br>17%, 8%<br>45%	1.7e-08,Yes, 0.17, [0.23,0.11]<br>1.4e-21,Yes, 0.28, [0.22,0.34]	0.337<br>0.014	11.6k, PASS, (0.213, 0.045), (0.12, -0.21)<br>16.4k, PASS, (0.732, 0.045), (-0.03, -0.16)<br>NA	15, 20<br>-0.015, 0.014, -0.008			
Menarche Age [2714]<br>Questionnaire/Cognitive<br>Age When Periods Started (menarche)<br>NA	85.6k, 5k,<br>18%, 3%<br>51%	0.052,Yes, 0.07, [0.14,-0.0]<br>1.3e-07,Yes, 0.18, [0.11,0.25]	0.015<br>0.054	7.4k, PASS, (0.013, 0.348), (0.26, -0.11)<br>11.2k, FAIL, (0.003, 0.244), (0.28, 0.11)<br>NA	0, 0<br>-0.014, 0.023, 0			
Menopause Age [3581]<br>Questionnaire/Cognitive<br>Age At Menopause<br>NA	59.4k, 2k,<br>12%, 2%<br>35%	0.035,Yes, 0.1, [0.19,0.02]<br>1.1e-14,Yes, 0.36, [0.27,0.45]	0.623<br>0.821	4.2k, PASS, (0.062, 0.73), (0.33, -0.05)<br>5.3k, PASS, (0.259, 0.819), (0.17, -0.03)<br>NA	1, 1<br>-0.044, 0.041, 0.013			
Monocyte % [30190]<br>Biomarkers<br>Monocyte Percentage<br>Monocytes [#/v] in Blood by Automated count	129k, 10k,<br>16%, 9%<br>39%	2.6e-16,Yes, 0.24, [0.3,0.18]<br>1.2e-48,Yes, 0.43, [0.37,0.48]	0.03<br>7.8e-04	11.7k, PASS, (5.3e-05, 0.001), (0.4, -0.34)<br>16.7k, PASS, (1.9e-04, 9.7e-07), (0.28, -0.35)<br>NA	4, 10<br>-0.019, 0, -0.004			
Monocytes [30130]<br>Biomarkers<br>Monocyte Count<br>Monocytes [#/v] in Blood by Automated count	129k, 10k,<br>18%, 7%<br>45%	7.8e-06,Yes, 0.13, [0.19,0.07]<br>1.8e-28,Yes, 0.33, [0.27,0.39]	0.387<br>0.002	11.7k, PASS, (0.007, 0.015), (0.26, -0.23)<br>16.7k, PASS, (4.5e-04, 0.703), (0.28, -0.03)<br>59.9k, FAIL, (0.325, 0.141), (0.04, -0.06)	6, 6<br>-0.022, 0, 0			
Neuroticism [20127]<br>Questionnaire/Cognitive<br>Neuroticism Score<br>NA	136k, 11k,<br>8%, 1%<br>28%	0.334,Yes, 0.03, [0.08,-0.03]<br>7.0e-07,Yes, 0.14, [0.08,0.19]	0.297<br>0.422	NA<br>14.8k, PASS, (1.1e-04, 0.104), (0.3, -0.15)<br>NA	0, 0<br>-0.006, -0.02, -0.011			
Neutrophill % [30200]<br>Biomarkers<br>Neutrophill Percentage<br>Neutrophils/100 leukocytes in Blood by AC	129k, 10k,<br>12%, 3%<br>32%	5.6e-13,Yes, 0.21, [0.26,0.15]<br>3.1e-06,Yes, 0.13, [0.08,0.19]	0.045<br>0.2	11.7k, PASS, (0.131, 5.2e-04), (0.15, -0.32)<br>16.8k, PASS, (0.093, 0.002), (0.13, -0.25)<br>53k, PASS, (0.01, 3.0e-05), (0.11, -0.19)	7, 8<br>-0.014, -0.031, -0.008			
Neutrophills [30140]<br>Biomarkers<br>Neutrophill Count<br>Neutrophils [#/v] in Blood by Automated count	129k, 10k,<br>14%, 4%<br>35%	9.6e-11,Yes, 0.18, [0.24,0.13]<br>1.7e-08,Yes, 0.16, [0.11,0.22]	0.084<br>0.401	11.7k, PASS, (0.7, 0.398), (0.04, -0.08)<br>16.7k, PASS, (0.226, 0.274), (-0.1, -0.08)<br>63.5k, FAIL, (0.007, 0.53), (-0.11, -0.02)	9, 7<br>-0.016, -0.021, -0.007			
Numeric Trail [20156]<br>Questionnaire/Cognitive<br>Duration To Complete Numeric Path (trail #1)<br>NA	56.7k, 1k,<br>5%, 1%<br>31%	0.206,Yes, 0.06, [0.16,-0.04]<br>0.027,Yes, -0.11, [-0.21,-0.01]	0.554<br>0.284	NA<br>2.3k, PASS, (0.005, 0.261), (0.73, 0.27)<br>NA	0, 0<br>-0.062, 0, 0			
Outdoor Time [1050]<br>Questionnaire/Cognitive<br>Time Spend Outdoors In Summer<br>NA	152k, 14k,<br>5%, 1%<br>29%	0.056,Yes, 0.05, [0.1,-0.0]<br>0.118,Yes, 0.04, [-0.01,0.09]	0.265<br>0.376	13k, PASS, (0.123, 0.831), (0.12, 0.02)<br>18.6k, FAIL, (8.9e-13, 0.044), (0.53, 0.14)<br>NA	0, 0<br>-0.016, 0.01, 0			

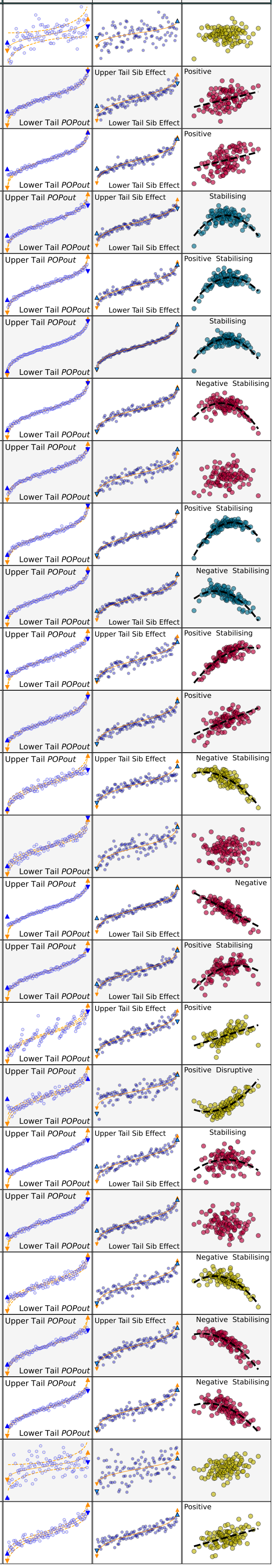

Supplementary Table 1: Traits 25-50

bioRxiv preprint doi: https://doi.org/10.1101/2024.11.18.624155; this version posted November 20, 2024. The copyright holder for this preprint (which was not certified by peer review) is the author/funder, who has granted bioRxiv a license to display the preprint in perpetuity. It is made available under aCC-BY 4.0 International license.

Trait Info	Trait Statistics	*POPout* Tests (Upper Tail / Lower Tail)	Sib Tests (Upper / Lower)	Replication	Rare/Selection
Abbreviated Name [UKB ID],<br>UKB Category,<br>UKB Name,<br>(All of Us Name)	Samples,Sib Pairs<br>SNP-h^2,PRS-r^2<br>Sib-h^2	Pval, 5% FDR, Effect Size, 95% CI	Meta<br>P-values	Repeated Measures | Multi-Ancestry | All Of Us<br>Target Size, QC, pvals, effect sizes (Lower,Upper)	Rare Variants:<br>(Lower, Upper),<br>Model Params:<br>($Y \sim Xo + \beta X + \gamma X^2$)
PW Notch [4199]<br>Physical Measures<br>Position Of Pulse Wave Notch<br>NA	55.3k, 3k,<br>6%, 1%<br>17%	1.1e-04,Yes, 0.16, [0.24,0.08]<br>0.74,Yes, 0.01, [-0.07,0.09]	0.025<br>0.272	3.6k, PASS, (0.911, 0.292), (-0.02, 0.15)<br>12.5k, PASS, (0.82, 0.94), (0.02, 0.01)<br>NA	0, 0<br>-0.044, -0.017, 0
Phosphate [30810]<br>Biomarkers<br>Phosphate<br>Phosphate [Mass/v] in Serum or Plasma	121k, 9k,<br>9%, 3%<br>25%	0.085,Yes, 0.05, [0.11,-0.01]<br>0.158,Yes, 0.03, [-0.03,0.08]	0.465<br>0.56	8.6k, PASS, (0.004, 0.385), (0.09, -0.1)<br>15.9k, FAIL, (0.306, 0.749), (0.07, -0.03)<br>32.2k, PASS, (0.033, 0.013), (0.12, -0.14)	2, 4<br>-0.022, 0, 0
Platelet Crit [30090]<br>Biomarkers<br>Platelet Crit<br>Platelets [#/v] in Blood by Automated count	130k, 10k,<br>21%, 10%<br>52%	2.7e-16,Yes, 0.26, [0.31,0.2]<br>1.1e-50,Yes, 0.36, [0.3,0.41]	5.8e-04<br>2.2e-04	8.3k, PASS, (4.2e-04, 0.696), (0.42, -0.04)<br>16.8k, PASS, (2.2e-05, 0.123), (0.34, -0.13)<br>NA	9, 16<br>-0.022, -0.017, 0
Pred FEV % [20154]<br>Physical Measures<br>Forced Expiratory Volume In 1s Predicted Percentage<br>NA	98.2k, 6k,<br>16%, 3%<br>38%	0.39,Yes, 0.03, [0.09,-0.04]<br>2.7e-12,Yes, 0.24, [0.17,0.31]	0.291<br>0.043	NA<br>569, PASS, (0.152, 0.268), (0.41, 0.37)<br>NA	0, 0<br>-0.004, 0.015, 0
Pulse Rate [4194]<br>Physical Measures<br>Pulse Rate<br>Computed heart rate (mean)	51.2k, 3k,<br>10%, 1%<br>39%	9.2e-04,Yes, 0.14, [0.23,0.06]<br>0.728,Yes, -0.01, [-0.1,0.07]	0.044<br>0.406	3.3k, PASS, (0.523, 0.622), (0.12, 0.07)<br>11.7k, PASS, (0.283, 0.773), (-0.1, -0.03)<br>127k, PASS, (0.337, 0.004), (0.03, -0.08)	2, 1<br>-0.034, -0.031, -0.011
RBC Dist. Width [30070]<br>Biomarkers<br>Red Blood Cell Dist Width<br>Erythrocyte distribution width [Ratio] by AC	129k, 10k,<br>18%, 6%<br>41%	5.7e-60,Yes, 0.5, [0.55,0.44]<br>0.006,Yes, -0.08, [-0.13,-0.02]	4.1e-04<br>0.776	11.8k, FAIL, (0.228, 3.4e-06), (0.11, -0.39)<br>16.6k, PASS, (0.66, 6.9e-06), (0.04, -0.38)<br>65.1k, PASS, (0.017, 2.3e-10), (0.1, -0.25)	31, 5<br>0.005, -0.009, 0
RBCs [30010]<br>Biomarkers<br>Red Blood Cell Count<br>Erythrocytes [#/v] in Blood by AC	130k, 10k,<br>19%, 7%<br>51%	0.306,Yes, -0.03, [0.03,-0.08]<br>9.5e-21,Yes, 0.28, [0.22,0.33]	0.258<br>0.005	11.8k, PASS, (0.002, 0.643), (0.28, -0.04)<br>16.8k, PASS, (0.535, 0.115), (0.25, -0.11)<br>80.1k, PASS, (1.0e-15, 0.288), (0.3, 0.04)	13, 13<br>-0.016, 0.023, -0.006
Reticulocyte % [30240]<br>Biomarkers<br>Reticulocyte Percentage<br>Reticulocytes [#/volume] in Blood	127k, 10k,<br>16%, 5%<br>40%	2.9e-05,Yes, 0.12, [0.17,0.06]<br>0.004,Yes, 0.08, [0.02,0.13]	0.263<br>0.659	11.6k, PASS, (0.009, 0.331), (0.24, -0.08)<br>16.4k, PASS, (0.346, 0.419), (0.07, -0.07)<br>NA	21, 4<br>-0.015, -0.008, -0.007
Reticulocytes [30250]<br>Biomarkers<br>Reticulocyte Count<br>NA	127k, 10k,<br>16%, 5%<br>41%	1.6e-04,Yes, 0.11, [0.16,0.05]<br>0.046,Yes, 0.06, [0.0,0.11]	0.18<br>0.529	11.6k, PASS, (0.01, 0.699), (0.23, -0.03)<br>16.4k, PASS, (0.198, 0.307), (0.1, -0.09)<br>NA	21, 8<br>-0.014, 0.015, -0.008
SD Matches [20159]<br>Questionnaire/Cognitive<br>Number Of Symbol Digit Matches Made Correctly<br>NA	51.6k, 1k,<br>11%, 1%<br>39%	0.01,Yes, -0.12, [-0.03,-0.21]<br>0.003,Yes, 0.15, [0.05,0.25]	0.779<br>0.522	NA<br>2.8k, PASS, (0.832, 0.209), (0.04, 0.21)<br>NA	0, 0<br>-0.054, 0, 0
SHBG [30830]<br>Biomarkers<br>Shbg<br>NA	120k, 9k,<br>16%, 8%<br>50%	1.1e-32,Yes, 0.36, [0.42,0.3]<br>0.07,Yes, 0.05, [-0.0,0.1]	0.15<br>0.506	8.4k, PASS, (0.028, 0.004), (0.28, -0.3)<br>15.8k, PASS, (0.967, 0.002), (0.0, -0.26)<br>NA	6, 8<br>-0.011, -0.025, -0.011
Sitting Height [20015]<br>Physical Measures<br>Sitting Height<br>NA	167k, 17k,<br>29%, 13%<br>74%	4.3e-25,Yes, 0.27, [0.32,0.22]<br>3.5e-60,Yes, 0.44, [0.39,0.49]	3.3e-08<br>1.4e-04	15.1k, PASS, (0.009, 0.923), (0.21, -0.11)<br>22.4k, PASS, (6.6e-06, 1.5e-04), (0.31, -0.28)<br>NA	10, 23<br>-0.008, 0.015, -0.013
Sodium In Urine [30530]<br>Biomarkers<br>Sodium In Urine<br>Sodium [Moles/v] in Urine	163k, 16k,<br>5%, 1%<br>16%	2.1e-05,Yes, 0.11, [0.16,0.06]<br>0.051,Yes, -0.04, [-0.09,0.01]	0.569<br>0.087	14.2k, FAIL, (0.478, 0.354), (0.06, -0.08)<br>21.8k, PASS, (0.006, 0.631), (-0.19, 0.03)<br>3.6k, PASS, (0.761, 0.744), (0.05, 0.05)	0, 0<br>-0.012, 0, -0.008
Sphered Cell Vol [30270]<br>Biomarkers<br>Mean Sphered Cell Volume<br>NA	128k, 10k,<br>18%, 8%<br>47%	1.1e-08,Yes, 0.16, [0.21,0.11]<br>8.6e-06,Yes, 0.13, [0.07,0.18]	0.21<br>0.066	8.2k, PASS, (0.11, 0.011), (0.13, -0.28)<br>16.4k, FAIL, (0.054, 0.063), (0.16, 0.15)<br>NA	16, 17<br>-0.016, -0.032, -0.006
TV Watching [1070]<br>Questionnaire/Cognitive<br>Time Spent Watching Television<br>NA	158k, 15k,<br>8%, 1%<br>45%	0.01,Yes, 0.06, [0.11,0.01]<br>8.3e-04,Yes, 0.09, [0.04,0.14]	0.061<br>0.042	14k, PASS, (0.708, 0.544), (0.03, -0.06)<br>20.7k, PASS, (1.8e-04, 0.805), (0.26, -0.02)<br>NA	0, 0<br>-0.026, -0.023, 0
Testosterone [30850]<br>Biomarkers<br>Testosterone<br>Testosterone [M/v] in Serum or Plasma	121k, 9k,<br>7%, 2%<br>18%	7.5e-04,Yes, -0.1, [-0.04,-0.16]<br>0.049,Yes, -0.06, [-0.11,-0.0]	0.454<br>0.216	9.2k, PASS, (0.474, 0.438), (-0.08, 0.09)<br>15.9k, FAIL, (0.195, 0.019), (0.1, -0.19)<br>8.6k, PASS, (0.152, 0.008), (0.18, -0.28)	0, 4<br>-0.01, -0.009, -0.009
Time To Answer [4288]<br>Questionnaire/Cognitive<br>Time To Answer<br>NA	55.7k, 3k,<br>6%, 1%<br>26%	3.4e-04,Yes, 0.16, [0.24,0.07]<br>0.364,Yes, 0.04, [-0.04,0.12]	0.634<br>0.33	3.6k, PASS, (0.196, 0.464), (-0.23, -0.15)<br>12.3k, PASS, (0.455, 0.941), (-0.07, -0.01)<br>NA	0, 0<br>-0.044, -0.04, 0
Total Protein [30860]<br>Biomarkers<br>Total Protein<br>Protein [M/v] in Serum or Plasma	121k, 9k,<br>14%, 3%<br>36%	6.7e-05,Yes, 0.11, [0.17,0.06]<br>0.225,Yes, 0.04, [-0.02,0.09]	0.078<br>0.245	8.6k, PASS, (0.006, 0.422), (0.09, -0.1)<br>15.9k, PASS, (0.432, 0.06), (0.06, -0.15)<br>79.9k, PASS, (7.1e-08, 0.335), (0.2, -0.03)	1, 7<br>-0.015, -0.009, -0.007
Triglycerides [30870]<br>Biomarkers<br>Triglycerides<br>Triglycerides [M/v] in Serum or Plasma	133k, 11k,<br>15%, 4%<br>41%	0.028,Yes, -0.06, [-0.01,-0.11]<br>1.5e-07,Yes, 0.15, [0.09,0.2]	0.21<br>0.298	10.6k, PASS, (0.073, 0.235), (0.18, 0.12)<br>17.3k, FAIL, (0.074, 0.512), (0.14, 0.05)<br>63.1k, PASS, (0.643, 0.655), (0.02, 0.02)	9, 11<br>-0.022, 0.015, 0
Urea [30670]<br>Biomarkers<br>Urea<br>Urea nitrogen [M/v] in Serum or Plasma	133k, 11k,<br>8%, 2%<br>27%	0.028,Yes, 0.06, [0.11,0.01]<br>2.4e-06,Yes, 0.13, [0.07,0.18]	0.201<br>0.662	10.6k, PASS, (0.594, 0.995), (0.05, 0.0)<br>17.4k, PASS, (0.079, 0.164), (0.12, -0.11)<br>82.7k, PASS, (0.006, 7.4e-15), (0.1, -0.28)	5, 0<br>-0.012, 0.01, -0.01
Vitamin D [30890]<br>Biomarkers<br>Vitamin D<br>25-hydroxyvitamin D3 [M/v] in Serum or Plasma	126k, 10k,<br>7%, 2%<br>31%	0.043,Yes, -0.07, [-0.02,-0.12]<br>4.6e-10,Yes, 0.18, [0.12,0.23]	0.429<br>0.708	9.8k, PASS, (0.027, 0.828), (0.2, -0.02)<br>16.2k, PASS, (0.253, 0.435), (0.09, 0.06)<br>26.8k, PASS, (0.04, 0.123), (0.12, -0.09)	4, 3<br>-0.01, 0.021, -0.011
WBCs [30000]<br>Biomarkers<br>White Blood Cell Count<br>Leukocytes [#/v] in Blood by AC	130k, 10k,<br>17%, 4%<br>40%	1.0e-05,Yes, 0.12, [0.18,0.07]<br>0.019,Yes, 0.06, [0.01,0.12]	0.013<br>0.284	11.8k, PASS, (0.95, 0.291), (0.01, -0.11)<br>16.8k, PASS, (0.855, 0.344), (0.01, -0.07)<br>77.8k, PASS, (0.031, 4.2e-09), (0.08, -0.2)	11, 6<br>-0.017, -0.009, -0.005
Waist Circum [48]<br>Physical Measures<br>Waist Circumference<br>Waist Circumference (Mean)	167k, 17k,<br>14%, 3%<br>42%	0.014,Yes, -0.06, [-0.01,-0.1]<br>1.8e-08,Yes, 0.14, [0.09,0.18]	4.5e-04<br>0.052	15.5k, PASS, (0.003, 0.286), (0.24, 0.08)<br>22.5k, PASS, (0.021, 0.018), (0.15, 0.16)<br>NA	1, 0<br>0.006, 0.032, -0.027
Weight [21002]<br>Physical Measures<br>Weight<br>Weight (Mean)	167k, 17k,<br>20%, 6%<br>58%	0.726,Yes, -0.01, [0.04,-0.06]<br>1.1e-10,Yes, 0.15, [0.11,0.2]	0.488<br>0.497	15.5k, PASS, (0.002, 0.869), (0.25, 0.01)<br>22.5k, PASS, (2.5e-05, 0.009), (0.26, 0.18)<br>127k, PASS, (3.2e-04, 5.3e-05), (0.1, -0.11)	4, 1<br>0.006, 0.035, -0.028

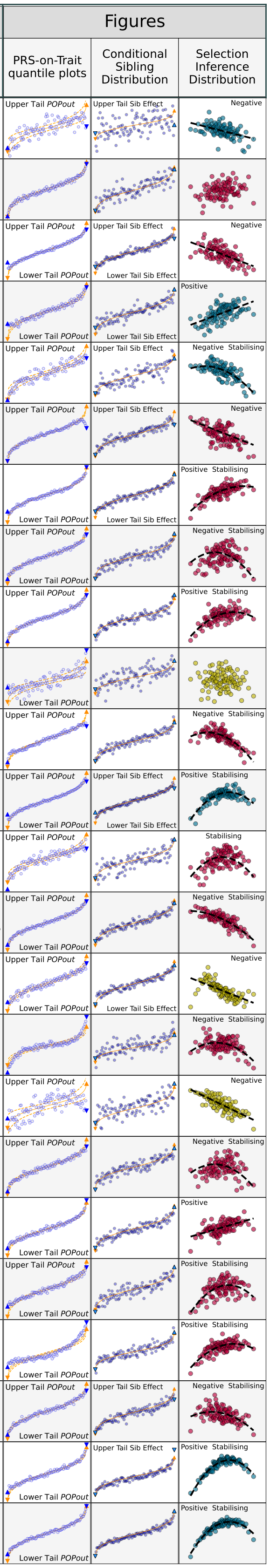

Supplementary Table 1: Traits 50-74
